# Enhanced Notch3 signaling contributes to pulmonary emphysema in a Murine Model of Marfan syndrome

**DOI:** 10.1038/s41598-020-67941-3

**Published:** 2020-07-02

**Authors:** Kathryn Jespersen, Zhibo Liu, Chenxin Li, Paul Harding, Kylie Sestak, Rishi Batra, Christopher A. Stephenson, Ryan T. Foley, Harrison Greene, Trevor Meisinger, B. Timothy Baxter, Wanfen Xiong

**Affiliations:** 1 Department of Surgery, 987690 Nebraska Medical Center, University of Nebraska Medical Center, Omaha, NE 68198-790 USA; 2 Department of Cardiothoracic Surgery, The First Affiliated Hospital of Nanchang University, Nanchang, 330006 Jiangxi China

**Keywords:** Developmental biology, Respiratory tract diseases

## Abstract

Marfan syndrome (MFS) is a heritable disorder of connective tissue, caused by mutations in the fibrillin-1 gene. Pulmonary functional abnormalities, such as emphysema and restrictive lung diseases, are frequently observed in patients with MFS. However, the pathogenesis and molecular mechanism of pulmonary involvement in MFS patients are underexplored. Notch signaling is essential for lung development and the airway epithelium regeneration and repair. Therefore, we investigated whether Notch3 signaling plays a role in pulmonary emphysema in MFS. By using a murine model of MFS, fibrillin-1 hypomorphic mgR mice, we found pulmonary emphysematous-appearing alveolar patterns in the lungs of mgR mice. The septation in terminal alveoli of lungs in mgR mice was reduced compared to wild type controls in the early lung development. These changes were associated with increased Notch3 activation. To confirm that the increased Notch3 signaling in mgR mice was responsible for structure alterations in the lungs, mice were treated with *N*-[*N*-(3,5-difluorophenacetyl)-l-alanyl]-*S*-phenylglucine *t*-butyl ester (DAPT), a $\gamma$-secretase inhibitor, which inhibits Notch signaling. DAPT treatment reduced lung cell apoptosis and attenuated pulmonary alteration in mice with MFS. This study indicates that Notch3 signaling contributes to pulmonary emphysema in mgR mice. Our results may have the potential to lead to novel strategies to prevent and treat pulmonary manifestations in patients with MFS.

## Introduction

Marfan syndrome (MFS) is an autosomal dominant inherited disorder of connective tissue that exhibits variable expressivity among affected individuals^1,2^. It is a systemic disorder with increased early morbidity and mortality related to aortic pathology. It is reported that 63% of MFS patients have an alteration in lung function^3^. Pulmonary emphysema appears to be a significant problem in those affected with neonatal MFS, the most severe form of MFS^4–6^. MFS is primarily caused by mutations in the *FBN1* gene, which encodes the extracellular matrix (ECM) protein fibrillin-1^7,8^. Fibrillin-1 is the main component of elastic tissue microfibrils, which form a scaffolding network for tropoelastin deposition but also interact with Notch, Notch ligands, and transforming growth factor β (TGF-β)^9,10^. Previous studies have shown histological and mechanical impairment of lungs in murine models of MFS, *Fbn1*^*mgΔ/mgΔ*^, *Fbn1*^*mgR/mgR*^ (mgR), and *Fbn1*^*C1039G/*+^^10,11^. Neptune et al. demonstrated that dysregulation of TGF-β activation contributed to the development of pulmonary emphysema in *Fbn1*^*mgΔ/mgΔ*^ and mgR mice^10^. The *Fbn1*^*mgΔ/mgΔ*^ mouse model represents a severe case of MFS; these mice produce approximately 10% of normal fibrillin-1. Because they are severely affected, *Fbn1*^*mg/mgΔ*^ mice die of cardiovascular complications around 2 weeks^12^. However, mgR represents a hypomorphic mutation of *FBN1*, and homozygous mgR mice express approximately 20% of normal fibrillin-1. These mice develop all the characteristic phenotypes in the skeletal, vascular, and pulmonary systems observed in classical MFS patients^13^.

Notch signaling is essential for lung development and is required for maintaining the integrity of both the epithelial and smooth muscle layers of the distal airway^14–16^. Notch genes encode large single-transmembrane receptors responsible for mediating communication between neighboring cells; this communication is crucial to direct cell fate decisions during organ development^15^. Four Notch receptors have been identified in mammals: Notch1, Notch2, Notch3, and Notch4. They are composed of a large extracellular domain, which mediates ligand interaction; a transmembrane domain; and an intracellular domain. In the canonical signaling pathway, Notch receptors are proteolytically cleaved by several proteinases upon receptor-ligand binding at the cell surface. One major proteinase responsible for Notch cleavage is γ-secretase^15^. Proteolytic cleavage of Notch releases the Notch intracellular domain (NICD) and allows it to translocate to the nucleus, where it interacts with transcription factor CBF-1 to promote transcriptional activation of downstream effectors.

Notch signaling plays a critical role in differentiation, regeneration, and repair of the airway epithelium^16–23^. In the airway epithelium of the developing embryo, Notch signaling maintains a balance between ciliated, secretory, and neuroendocrine cells ^17,18^. Constitutive Notch signaling inhibits the differentiation of alveolar epithelium^16^. Postnatally, Notch signaling in the epithelium is responsible for a host of processes involved in development of the distal lung^19^. It also prevents epithelial club cells from differentiating into goblet cells and plays a critical role in recovery of the airway epithelium following injury^20–22^. Of the four Notch receptors, Notch3 has been found to play the most critical role in regulating alveolar epithelium. A gain of function in Notch interferes with distal alveolar formation by disrupting differentiation processes^16^. Constitutive Notch3 expression in the peripheral epithelium inhibits type II pneumocytes from differentiation into type I pneumocytes; this results in an altered lung morphology^23^. Histological and functional changes were observed in both lung development and maturation of patients and a murine model of MFS^10^. However, the mechanism by which Notch3 exerts its function in pulmonary emphysema in MFS remains unknown.

To investigate the influence of Notch signaling in MFS alveolar development, we studied the role of Notch3 on pulmonary morphogenesis in MFS mice. Here we show the pulmonary emphysematous changes were observable at a very early stage in the development of MFS mice. This change is correlated with increased Notch3 activation. We found that inhibition of Notch signaling rescued development of the distal alveoli in MFS mice.

## Results

### Distal airspace was progressively increased in the lungs of mgR mice

**Figure 1:**
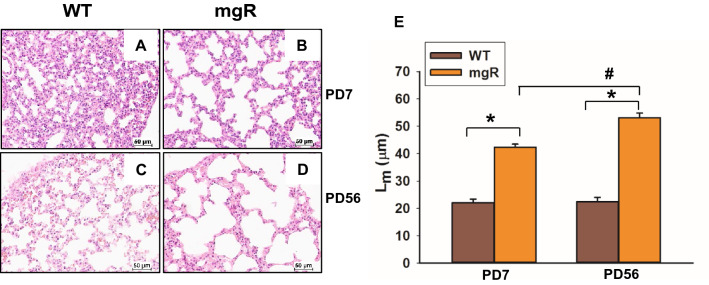
Morphometric analysis of distal lung tissue shows an increase in airspace in mgR mice. (**A**–**D**) Lung tissue from wild type (WT) and Marfan (mgR) mice were sectioned and stained with hematoxylin and eosin (H&E) at the time points of PD7 and PD56. Representative sections from the 8–10 mice are presented. (**E**) The mean linear intercept (L_m_) analysis of lung tissue samples from WT and mgR mice. *P < 0.01 compared to WT control. ^#^P < 0.01 compared to mgR mice at PD7, ANOVA with Tukey–Kramer post hoc test.

Disorders of the respiratory system have been noted in a distinct subgroup of patients with MFS. Previous studies of mouse models of Marfan syndrome (*Fbn1*^mgΔ/mgΔ^ and *Fbn1*^mgR/mgR^) have shown that *Fbn1*^mgΔ/mgΔ^ and *Fbn1*^mgR/mgR^ mice developed pulmonary emphysema as early as postnatal day (PD)1 and PD14, respectively^10^. Because they are severely affected, *Fbn1*^mgΔ/mgΔ^ mice have a short lifespan, 2–3 weeks, which makes it hard to study the pathogenesis of emphysema and test pharmacologic agents. For this reason, we used a widely accepted murine model of MFS (*Fbn1*^mgR/mgR^ or mgR) which is less severely affected allowing later assessment of interventions. Histological sections of lungs were analyzed by H&E staining. Compared to their wild type (WT) littermates, lungs of mgR mice had enlarged distal airspaces at PD7 and PD56 (Fig. 1B, D) which is consistent with previous findings^10^. The mean linear intercept (L_m_), as a measure of interalveolar wall distance, was significantly increased in mgR mice compared with WT littermate controls (Fig. 1E). The progressive distal airspace enlargement was seen between PD7 and PD56 in mgR mouse lungs.

### Notch3 signaling was increased in the lungs of mgR mice

**Figure 2:**
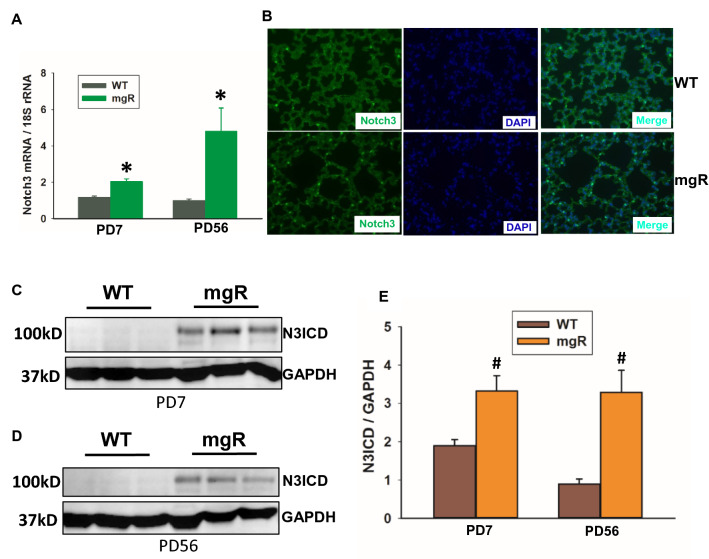
Notch3 level and activation were increased in the lungs of mgR mice. (**A**) qRT-PCR analysis for Notch3 in the lungs of WT and mgR mice at the time points of PD7 and PD56 (n = 5–10/group). (**B**) Representative images of immunofluorescence staining of Notch3 (green) expression in the lungs of WT and mgR mice at PD56 (n = 5/group), nuclei (DAPI). (**C**–**E**) Western blot analysis of Notch3 activation. Protein from mouse lung of WT (n = 18) and mgR (n = 12) mice at PD7 and WT (n = 8) and mgR (n = 8) at PD 56 was extracted. Western blot analysis was performed. The bar graph (**E**) shows relative active Notch3 (N3ICD) levels in the lung tissue. Values are expressed as mean ± SEM. *P < 0.05, ^#^P < 0.01, compared to WT controls. Student’s *t* test.

Notch signaling is essential for lung development. We examined Notch1-4 mRNA levels by real-time PCR. No notable difference in mRNA expression of Notch1, 2, and 4 was observed between WT and mgR mice (Supplemental Fig. 1). However, the expression of Notch3 mRNA was significantly increased in the lungs of mgR mice at PD7 and PD56 (Fig. 2A). The increased Notch3 expression in the lung of mgR mice was confirmed with immunofluorescence staining (Fig. 2B). Constitutive activation of Notch3 resulted in altered lung morphology and inhibition of pneumocyte differentiation and proliferation^23^. We examined Notch3 activation using Western blot analysis. As shown in Fig. 2C–E, the levels of active Notch3 (Notch3 intracellular domain, N3ICD) in mgR mice were increased in both PD7 and PD56. These data don’t directly implicate Notch3 in emphysema but identify a putative therapeutic target we believe to have a role in lung development.

**Figure 3:**
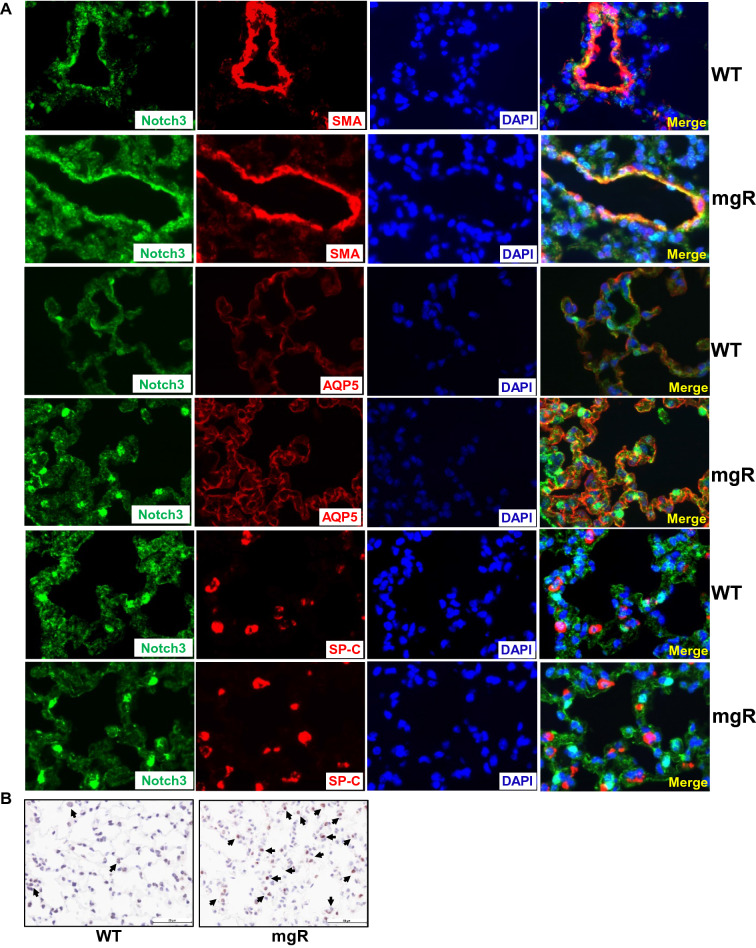
Notch3 production in smooth muscle cells (SMCs) and alveolar epithelial cells (AECs) was increased in the lung of mgR mice. (**A**) Distal lung sections from WT and mgR mice (n = 4–5/group) were subjected to immunofluorescence staining. Notch3 expression in the lungs was detected by Notch3 immunolabeling (green). SMCs, AEC-I, and AEC-II were detected by SMA, AQP5, and SP-C immunolabeling (red), respectively. Nuclei were counterstained with DAPI. Co-localization of Notch3 and SMA, AQP5, or SP-C was shown by the yellow color. (**B**) Lung tissue sections from WT and mgR mice at PD7 were immunostained with caspase3 (CC3) antibody. Arrows indicate CC3-positive stained cells.

Since Notch3 is involved in proliferation and differentiation of alveolar epithelial cells (AEC) and vascular smooth muscle cells (SMCs)^23–25^, we characterized which cell types were responsible for high production of Notch3 in mgR mice using immunofluorescence staining of lungs from WT and mgR mice at PD56 (Fig. 3A). Notch3 was co-expressed with SMC marker (SMA), type I AEC (AEC-I) marker (AQP5), and type II AEC (AEC-II) marker (SP-C) (Fig. 3A). However, there was more intense staining of Notch3 in the SMCs and AEC-I of mgR mice compared to WT controls. We further assessed cell proliferation in the lungs of WT and mgR mice by immunostaining with Ki67, a proliferation marker. We found no significant difference between WT and mgR mice at PD7 and PD56 (Supplemental Fig. 2). It was reported that pneumocyte apoptosis contributed to emphysematous changes in the lungs of *Fbn1*^mgΔ/mgΔ^ mice^10^. We assessed cell apoptosis in the lungs of WT and mgR mice by immunostaining of caspase 3 (CC3), a cell apoptosis marker. We found more caspase 3-positive cells in the lungs of mgR mice compared to that of WT mice at PD7 (Fig. 3B).

### Inhibition of Notch activation remarkably reduced pulmonary emphysematous changes in mgR mice

**Figure 4:**
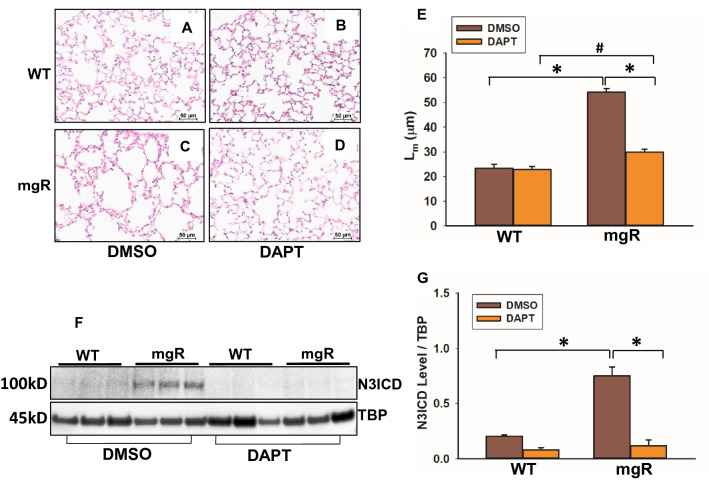
Blocking of Notch3 activation inhibited pulmonary alteration in mgR mice. WT and mgR mice were started treatment with DAPT, a ϒ-secretase inhibitor at PD10. Mice were sacrificed at PD56. Histological analyses of lungs were done with H&E staining. Representative sections from the 5 mice in each group are presented, WT mice treated with DMSO (**A**) and DAPT (**B**), mgR mice treated with DMSO (**C**) and DAPT (**D**). (**E**) The mean linear intercept (L_m_) analysis of lung tissue samples from DAPT- or DMSO-treated WT and mgR mice. *P < 0.001 compared to DMSO-treated mgR mice. ^#^P < 0.01 compared to DAPT-treated mgR mice, ANOVA with Tukey–Kramer post hoc test. (**F**) Active Notch3 levels in the nuclear fraction of lungs of DMSO- or DAPT-treated WT and mgR mice (n = 5/group) were examined by Western blot. Quantitation of N3ICD levels is shown in bar graph on the right (**G**). *P < 0.001 compared to DMSO-treated controls, ANOVA with Tukey–Kramer post hoc test.

To determine whether inhibition of Notch3 activation in mgR mice could prevent pulmonary morphological changes, we treated WT and mgR mice with ϒ-secretase inhibitor, DAPT, to inhibit Notch signaling. The treatment started at PD10 and mice were sacrificed at PD56. Histological changes of lung tissue were evaluated using H&E staining (Fig. 4A–D). Quantitation of changes in alveolar space was performed with the mean linear intercept analysis. DAPT treatment had no effect on WT mice but remarkably reduced distal airspace enlargement in mgR mice (Fig. 4E). Notch3 activation levels in lungs were examined by Western blot. Active Notch3 was significantly higher in DMSO-treated mgR mice compared to DMSO-treated controls, but lower in DAPT-treated mgR mice compared to DMSO treatment (Fig. 4F, G).

**Figure 5:**
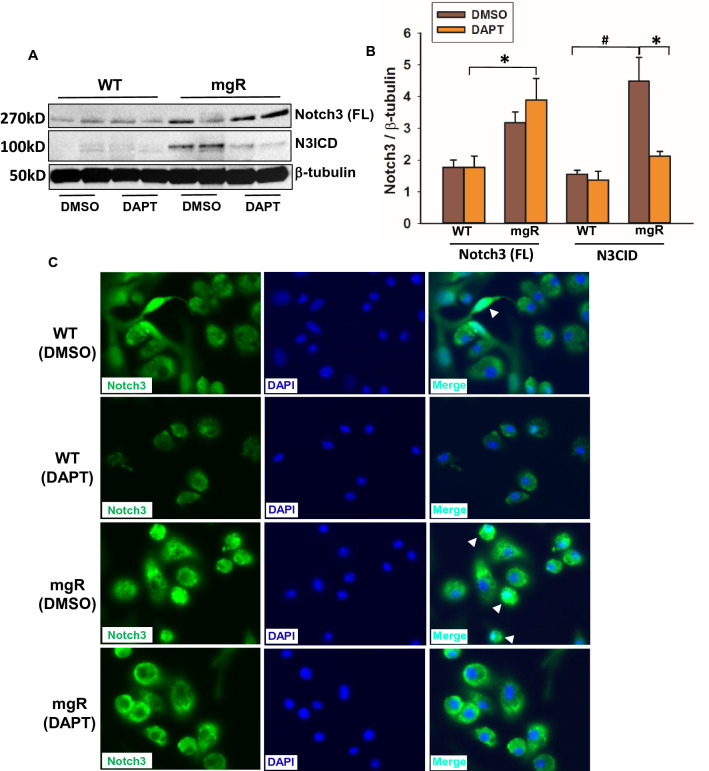
Notch3 activation was increased in lung cells of mgR mice. Epithelial cells were isolated from lungs of WT and mgR mice (n = 6/group). Cells from WT and mgR mice were treated with DAPT for 48 h. Notch3 activation and nuclear translocation were examined by Western blot (**A**) and immunofluorescence staining, respectively (**C**). Quantitation of N3ICD levels in the cells from Western blot analysis is shown in bar graph (**B**). *P < 0.05 compared to DAPT-treated cells. ^#^P < 0.001 compared to DMSO-treated WT cells, ANOVA with Tukey–Kramer post hoc test.

To further confirm the role of Notch3 activation in pneumocytes, lung cells were isolated from WT and mgR mice. AEC identity was confirmed with immunofluorescent staining (Supplemental Fig. 3). Cells were treated with DAPT for 48 h. As shown in Fig. 5A and B, cells from mgR mice had higher levels of active Notch3 compared with cells from WT mice. DAPT treatment significantly inhibited Notch3 activation in the cells from mgR mice. Furthermore, increased Notch3 translocation to nuclei (white arrowheads) was observed in DMSO-treated cells from mgR mice. DAPT treatment inhibited Notch3 nuclear translocation (Fig. 5C). These data suggest that increased activation of Notch3 in lung cells contributes to emphysematous changes in mgR mice. Inhibition of Notch3 signaling may attenuate pulmonary emphysema in MFS.

**Figure 6:**
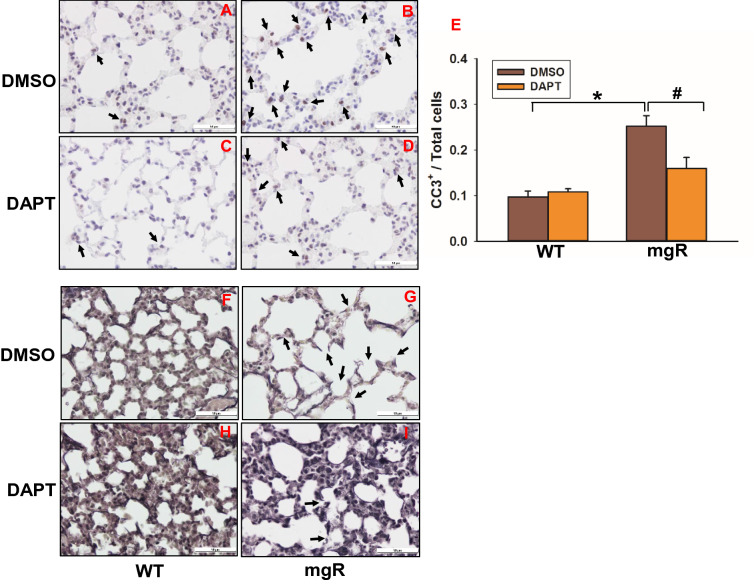
DAPT treatment inhibited cell apoptosis and attenuated elastin degradation in the lungs of mgR mice. (**A**–**D**) Lung tissue sections from DMSO (**A**, **B**) or DAPT (**C**, **D**) treated WT (**A**, **C**) and mgR (**B**, **D**) mice were immunostained with caspase3 (CC3) antibody. Quantitation of CC3^+^ cells is shown in bar graph on the right (**E**) (n = 4–5/group). #P < 0.05, *P < 0.001 compared to DMSO-treated mgR mice, ANOVA with Tukey–Kramer post hoc test. (**F**–**I**) VVG staining of elastic fiber in the lungs of WT (**F**, **H**) and mgR (**G**, **I**) mice treated with DMSO (**F**, **G**) or DAPT (**H**, **I**) (n = 5–10/group).

To determine whether inhibition of Notch activation signaling has effects on AEC apoptosis and elastin degradation in the lungs of mgR mice, we evaluated apoptosis of AECs in mgR mice with or without DAPT-treated mice using immunostaining of caspase 3 (Fig. 6A–D). We found fewer caspase3-positive cells in the lungs of DAPT-treated mgR mice compared to DMSO-treated mgR mice (Fig. 6D, E). VVG staining of lung tissue showed elastin degradation and fragmentation in the lungs of mgR mice (Fig. 6G). DAPT treatment preserved lung elastic fiber integrity in mgR mice (Fig. 6I). These results demonstrate that enhanced Notch3 activation stimulates AEC apoptosis and elastic fiber degradation in mgR mice.

## Discussion

Pulmonary dysfunction is one of the common manifestations in MFS. However, the pathogenesis and molecular mechanisms of pulmonary alterations in MFS are unknown. Notch3 signaling plays an important role in regulating the formation and development of alveolar epithelium. The purpose of the present study was to investigate the role of Notch signaling in the pathogenesis of pulmonary emphysema in MFS. By using mgR mice, a murine model of MFS, we have shown that emphysematous changes are apparent at an early age, PD7. The distal airspace enlargement was progressively increased with age. We have identified increased Notch3 activation in the lungs of mgR mice. Furthermore, Notch inhibition using a γ-secretase inhibitor, DAPT, attenuated lung morphological changes. These data suggest that enhanced Notch3 activation contributes to pulmonary morphological changes in mgR mice.

MFS is chiefly caused by mutations in the *FBN1* gene, which encodes for the extracellular matrix (ECM) glycoprotein fibrillin-1^26,27^. Fibrillin-1 is the main component of microfibrils, and it associates with elastin to form elastic fibers in the ECM. Currently, there are three commonly used murine models of MFS: *Fbn1*^*mgΔ/mgΔ*^, *Fbn1*^*mgR/mgR*^ (mgR), and *Fbn1*^*C1039G/*+^. *Fbn1*^*mgΔ/mgΔ*^ mice represent a severe form of the disease, expressing approximately 10% of normal fibrillin-1. These mice die of cardiovascular complications within 2–3 weeks of age^12^. The mgR model demonstrates a hypomorphic mutation of *FBN1*, and homozygous mice produce approximately 20% of normal fibrillin-1. These mice display clinical features and manifestations similar to classical MFS patients, and they die naturally at an average age of 3–6 months^13^. C1039G/+ is a heterozygous missense mutation of *FBN1*. *Fbn1*^*C1039G/*+^ mice show mild manifestations of MFS^27^. Previous studies showed that dysregulated TGF-β activation contributed to MFS pathogenesis^28,29^. Inhibiting TGF-β activation with a TGF-β-neutralizing antibody improved alveolar septation in the lungs of *Fbn1*^*mgΔ/mgΔ*^ mice^10^. However, this pharmacological inhibition of TGF-β underscores the complex and context-dependent roles of TGF-β in MFS. While previous studies investigating systemic neutralization of TGF-β in the *Fbn1*^*C1039G/*+^ model prevented the formation of thoracic aortic aneurysm (TAA)^28^, later studies using the mgR model demonstrated that TGF-β exerted an opposing effect on TAA pathology: TGF-β neutralization broadly correlated with both early- and late-stage TAA progression^30^. The authors showed that early (PD16) treatment with TGF-β-neutralizing antibodies exacerbated TAA formation, while later (PD45) treatment demonstrated a contrasting beneficial effect^30^. Our previous study supported that the initial consequence of *FBN1* mutation was not accompanied by a significant increase in TGF-β activation^31^. These contrasting examples of TGF-β contribution to aortic physiology in both early and late MFS stages provide a strong rationale for this study. The identification of new biomarkers independent of the TGF-β signaling pathway, such as Notch, is crucial to better understand the origin of pulmonary emphysema development in MFS.

Notch signaling plays a critical role in the development of the respiratory system^19^. Notch2 is the primary receptor involved in Clara/ciliated cell fate selection^32^. Notch1-3 are involved in regulating pulmonary neuroendocrine cell fate selection^14^. Notch4 is an endothelial cell-specific mammalian Notch gene^33^. In order to determine the role of Notch signaling in lung development and pulmonary impairment related to MFS, we examined the expression of Notch1-4 receptors in the lungs of mgR and WT mice. We found that Notch3 expression was significantly increased in the lungs of mgR mice. To isolate the role of Notch3 in MFS-related pulmonary morphological changes, we evaluated the histological differences between lungs of WT and mgR mice. Apparent structural alterations were observed in the lungs of 1-week-old mgR mice compared to WT controls. Alveolar septation was progressively reduced in mgR mice with age. This change is associated with increased Notch3 activation. Previous studies demonstrate that fibrillin-1 regulates Notch expression, and abnormal fibrillin-1 expression impacts Notch signaling^34^. Fibrillin-1 expression is critical for latent TGF-β binding protein (LTBP)-1, -3, and -4 to incorporate into lung tissue^35^. LTBP-1 deposition in the ECM was shown to be significantly decreased in association with fibrillin-1 knockdown in retinal epithelial cells^36^. LTBP-1 can directly bind to the Notch3 extracellular domain^37^. Therefore, we hypothesized that fibrillin-1 mutation leads to enhanced activation/signaling of Notch3 which in turn impairs lung development, specifically distal airspace septation. We administered DAPT, a γ-secretase inhibitor, to mgR mice starting at PD10. DAPT inhibits Notch3 signaling by preventing the proteolysis of Notch3. Histological analysis of the lungs in mgR mice at PD56 showed that DAPT treatment attenuated pulmonary alteration and suppressed the Notch3 activity in lung cells of mgR mice. Furthermore, fibrillin-1 deficiency stimulated lung cell apoptosis. Notch3 activation can induce the intrinsic apoptotic cascade^38^. Our results showed that inhibition of Notch3 by DAPT treatment reduced lung cell apoptosis and preserved the integrity of elastic fibers in the lungs of mgR mice. These results demonstrate that increased Notch3 activation in fibrillin-1 deficient mgR mice contributes to lung emphysematous changes by inducing apoptosis and elastin degradation. However, further studies are required to investigate potential crosstalk between Notch3 and fibrillin-1.

Several studies have suggested that in the mammalian lung, Notch signaling is linked to a variety of diseases, including chronic obstructive pulmonary disease (COPD), asthma, pulmonary fibrosis, and lung lesions in some congenital disease^23,39–41^. Notch3 has been show to play a role in regulating alveolar epithelium; specifically, its constitutive expression alters lung morphology by inhibiting type II pneumocytes from differentiating into type I pneumocytes^23^. Conversely, another study demonstrated that Notch3 was downregulated in both adult smokers and smokers with COPD^42^. These studies suggest that Notch3 is critical to maintain normal epithelial cell fate decision pathways in the airway epithelium. Differential thresholds of Notch3 signaling activation may determine normal or abnormal lung development. Our data demonstrate a direct link between enhanced Notch3 activation/signaling and impairment of distal alveolar septation. This pulmonary pathology can be attenuated with treatment of a drug to inhibit Notch signaling. Our results suggest that inhibition of Notch3 signaling in the MFS lung may prove to be an effective strategy in prevention and treatment of MFS-related pulmonary alterations.

## Methods

### Animals

All animal protocols in this study were reviewed and approved by the Institutional Animal Care and Use Committee for the University of Nebraska Medical Center (Permission number: 17-074-08FC). All experiments and procedures were performed in accordance with the regulations and guidelines set forth by the University of Nebraska Medical Center Animal Care Committee for the use and care of laboratory animals. Heterozygous mutant mice (*Fbn1*^*mgR/*+^ or mgR/+) in a mixed C57BL/6J;129 SvEv background were mated to generate homozygous *Fbn1* mutant mice (*Fbn1*^*mgR/mgR*^ or mgR) and wild type (WT) littermates. Mice were genotyped at postnatal day (PD)7 by PCR^43,44^. Because male and female mice were equally affected, both sexes were used in this study. WT littermates and homozygous mgR mice were sacrificed at PD7 (n = 20/group) and PD56 (n = 16/group). Typically these mice weigh 4–5 g at PD7 and 20–25 g at PD56. Mouse lungs were inflated and perfusion-fixed with 10% neutral buffered formalin^45^ or collected for RNA and protein extraction. For histological studies, the lower left lobe of the lungs was ligated, excised, immersed in formalin, and fixed for histological studies. In order to identify the role of Notch3, WT (n = 8/group) and mgR (n = 8/group) mice were treated with γ-secretase inhibitor, *N*-[*N*-(3,5-Difluorophenacetyl)-l-alanyl]-*S*-phenylglycine t-butyl ester (DAPT) (Abcam, Cambridge, UK). DAPT was suspended in DMSO. Mice were injected subcutaneously with 10 mg/kg of DAPT daily beginning at PD10 until sacrifice at PD56. A control group received DMSO only. After treatment, mice were sacrificed. Mouse lungs were perfusion-fixed for histology or collected for protein extraction. All mice were housed in the pathogen-free animal facility for the duration of the protocol.

### Histology and morphometric analysis

After 24 h fixation in formalin, the lungs were embedded in paraffin following our previous protocol^31^. Lung sections were cut and stained with hematoxylin for 1.5 min and eosin for 30 s (H&E) according to the manufacturer protocol (Abcam). Morphometric analyses utilized two 4-μm paraffin-embedded, H&E-stained sections of the lower left lobe from each of the 8–10 mice. Each section was subjected to mean linear intercept analysis according to previously published methods^46,47^. The mean linear intercept, a measure of interalveolar wall distance, was determined by light microscopy at a total magnification of × 100 and obtained by dividing the total length of a line drawn across each lung section by the total number of intercepts encountered in 20 lines per section^47^. As previously performed by our lab, mouse lungs were stained for connective tissue using Verhoeff solution, ferric chloride, sodium thiosulfate, and Van Gieson solution (BBC Biochemical, Mt. Vernon, WA)^48^. Staining cycles alternated between fixing and washing procedures, as described previously^48^. Each slide was examined and photographed using light microscopy (× 40; Nikon).

### RT-PCR assay

Total RNA from mouse lungs was extracted using TRIzol reagent (Thermo Fisher Scientific, Waltham, MA). As previously conducted by our lab^31,48^, RNA was reverse transcribed into cDNA using iScript Reverse Transcription Supermix (Bio-Rad Laboratories, Inc.). Real-time RT-PCR was performed using SsoAdvanced Universal SYBR® Green Supermix according to the manufacturer protocol (Bio-Rad Laboratories, Inc.) on an ABI StepOne machine (Thermo Fisher Scientific). The primer pairs used for assessing gene expression of Notch1, Notch2, Notch3, and Notch4 were as follows: 5′-CCCACTTTTAGCTCCAGCAG-3′ and 5′-AGGTTCTGCCACAAAACCAC-3′ (Notch1); 5′-GAGGATGAGGCTTTGCTGTC-3′ and 5′-GTTCTGCCTGAGGAGGAGTG-3′ (Notch2); 5′-CTCTGTGGTGATGCTGGAGA-3′ and 5′-AATCAAGTCGCTCCACTGCT-3′ (Notch3); 5′-CATCCCAGCCTATGACCAGT-3′ and 5′-CCTTCTGGTCTGCAGTCTCC-3′ (Notch4);

### Immunofluorescence and immunohistochemistry

Paraffin-embedded mouse lungs were serially sectioned at 4 μm. Tissue sections and cells on the slides were subsequently incubated with anti-Notch3 antibody (1:250) (Proteintec, Rosemont, IL) for 44 min at 37 °C, anti-rabbit HQ (Roche, South San Francisco, CA) for 16 min at 37 °C, anti-HQ horseradish peroxidase (HRP) (Roche) for 16 min at room temperature, and FITC kit (Roche) for 12 min. After denaturation of antibodies with CC2 solution for 8 min at 91 °C and neutralization of previously bound HRP conjugate for 12 min, sections were incubated with anti-AQP5 (1:500), SP-C (1:500), or SMA (1:100) antibodies (Abcam) for 44 min at 37 °C. Then sections were further incubated with anti-rabbit HQ for 16 min at 37 °C, anti-HQ HRP (Roche) for 16 min, and Cy5 kit (Roche) for 16 min and DAPI for 5 min at room temperature. Fluorescence images were captured with a Leica epifluorescence wide-filed microscope (North Central Instruments DMRXA2 Model) and CCD camera (Hamamatsu Photonics).

Immunohistochemical staining was performed with anti-Ki-67 antibody (Abcam) and anti-caspase3 (CC3) antibody (Abcam, ab5694) with a dilution of 1:200 for 32 min at 37 °C. Discovery ChromoMap DAB kit (Roche) was used for antigen localization and the slides were processed using Ventana Discovery Ultra instruments (Roche). Microscopic fields (40 × objective) of lungs were collected from each slide. Images were captured by Roche Ventana iScan HT (Roche). Definiens Tissue Studio software was used for quantification of staining area and density. The mean density of Ki67 or CC3 was determined for five regions of interest per lung section.

### Isolation and culture of pulmonary epithelial cells

The pulmonary epithelial cell isolation was done by following the methods from Jansing et al.^49^ with minor modifications. WT and mgR mice (n = 6/group) were anesthetized. The lungs were perfused with PBS via the right ventricle to remove red blood cells. Then, a 22G cannula was inserted into the trachea. The digestion buffer, dispase (5 units/ml) and DNase (0.01%) (Sigma), was instilled into the lungs through the cannula. After 5 min, the lungs were dissected and incubated with digestion buffer for an hour at 37 °C. The cells were washed with PBS. Erythrocytes were lysed with erythrocyte lysis buffer (Gibco, Gaithersburg, MD). The cells were washed again and re-suspended in lung epithelial cell specific medium (Gibco), 40% RPMI-1640, 40% LHC-9, 20% fetal bovine serum. The cells were cultured on bovine collagen-coated plates (Advanced Biomatrix, San Diego, CA). Cells were stained with CD326 (EpCAM) and AQP5 antibodies (Invitrogen, Waltham, MA) to confirm the purity of alveolar epithelial cells. To study notch signaling in lung cells, we treated cells from WT and mgR mice with 20 μM DAPT for 48 h. DMSO treatment was used as a control. After treatment, cells were harvested for protein extraction.

### Western blot

The right lungs were homogenized. The protein from the lung tissue and cells was extracted with RIPA lysis and extraction buffer (Thermo Scientifi, Waltham, MA). Nuclear protein from fresh lung tissues was isolated using Nuclear Extraction Kit (Abcam), according to the manufacturer’s protocol. The protein concentration was standardized with the Bio-Rad Protein Assay Dye Reagent Concentrate #5,000,006 (Bio-Rad Laboratories, Hercules, CA). Thirty-five to seventy μg of protein extracts were loaded into 4–20% Criterion TGX precast gels (Bio-Rad Laboratories). Following electrophoresis, the gel was transferred onto a 0.45 μm PVDF membrane (Bio-Rad Laboratories). The membrane was incubated overnight at 4 °C with antibodies directed against Notch3 (1:1,000), β-tubulin (1:1,000), GAPDH (1:1,000), or TBP (1:1,000) (Cell Signaling, Beverly, MA). Bound primary antibodies were detected with HRP-conjugated, species specific, secondary antibodies (1:1,000) (Cell Signaling) using the Clarity Western ECL system (Bio-Rad Laboratories). The quantification was done using NIH imageJ software and standardized by internal loading controls.

### Statistical analyses

Data are expressed as mean value ± the standard error (SE) of the mean. For continuous variables, if the data were normally distributed, the Student’s *t* test (comparison between two groups) or ANOVA with the appropriate post hoc test (comparison among groups of three or more) were used. Statistical significance was accepted at a P < 0.05.

## Supplementary information

Supplementary file1 (PDF 862 kb)
