# Supplementary material for: Enhanced Notch3 signaling contributes to pulmonary emphysema in a Murine Model of Marfan syndrome
Source: Sci Rep. 2020 Jul 2;10:10949. doi: 10.1038/s41598-020-67941-3 (PMC7331498; doi:10.1038/s41598-020-67941-3)
Supplement: Supplementary file 1 — Supplementary file1 (PDF 862 kb) [file 41598_2020_67941_MOESM1_ESM.pdf]

Supplementary Information

Enhanced Notch3 signaling contributes to pulmonary emphysema in a Murine Model of Marfan syndrome

Kathryn Jespersen[1*], Zhibo Liu[2*], Chenxin Li[1], Paul Harding[1], Kylie Sestak[1], Rishi Batra[1], Christopher A. Stephenson, Ryan T. Foley, Harrison Greene, Trevor Meisinger[1], B. Timothy Baxter[1], and Wanfen Xiong[1]

Supplemental Figure 1

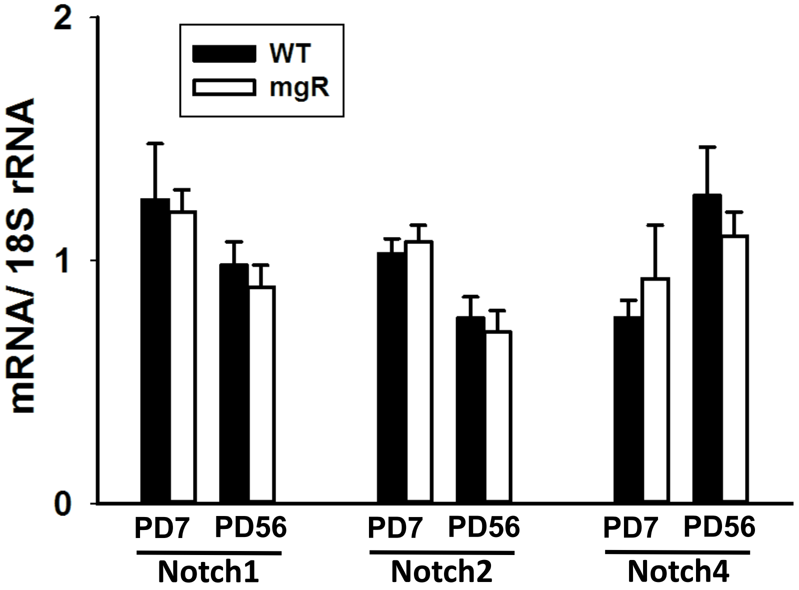

Supplemental Fig. 1. Quantitative real-time RT-PCR analysis for Notch1, 2, and 4 in the lungs of WT and mgR mice at the time points of PD7 and PD56 (n = 5-10/group).

Figure 2 Western blot

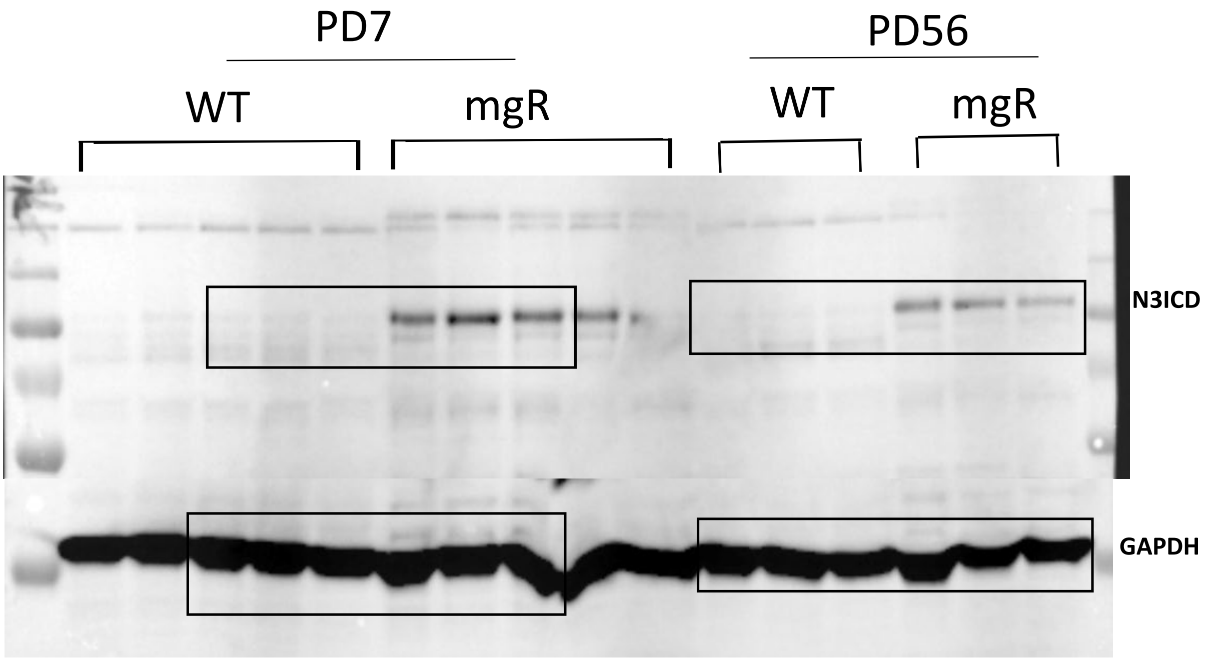

Figure 4 Western blot

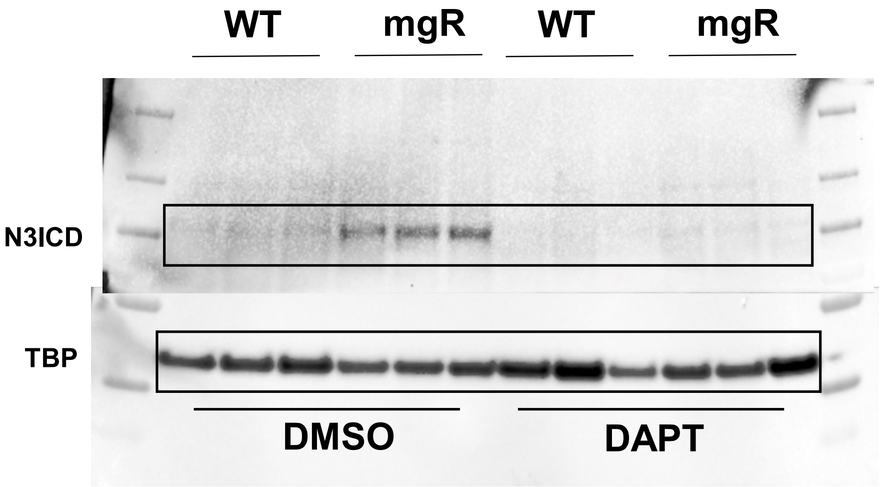

Figure 5 Western blot

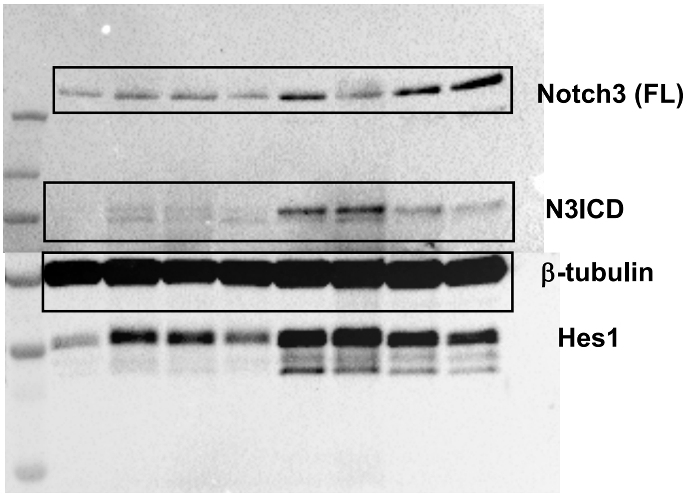

Supplemental Figure 2

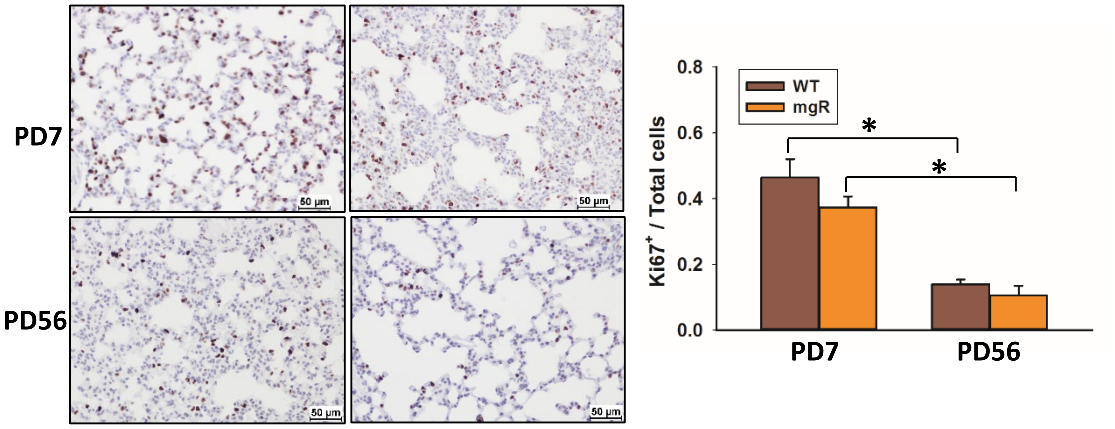

Supplemental Fig. 2. Lung cell proliferation in WT and mgR mice at PD7 and PD56 was analyzed by immunostaining with Ki67, a cell proliferation marker. Proliferative cells were quantitated by Definiens tissue studio software. There is no significant difference in cell proliferation between WT and mgR mice. However, as expected, higher cell proliferation rate occurs at PD7 compared to PD56 (n = 5 – 10/group). *P < 0.001 compared to PD56. ANOVA with Tukey-Kramer post hoc test.

Supplemental Figure 3

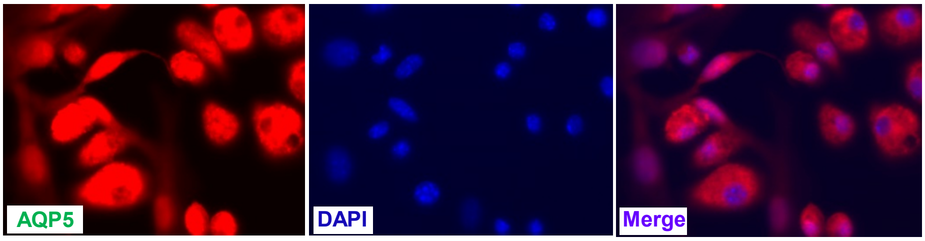

Supplemental Fig.3. Identification of alveolar epithelial cells isolated from mouse lung. Pneumocytes were isolated from mouse lungs. Cells were cultured and then immunostained with AQP5 antibody(red). Nuclei were counterstained with DAPI. Representative images indicate the majority cells isolated from mouse lungs are alveolar epithelial cells.
